# Protocol for the reconstruction of micromammals from fossils. Two case studies: The skulls of *Beremendia fissidens* and *Dolinasorex glyphodon*

**DOI:** 10.1371/journal.pone.0213174

**Published:** 2019-03-20

**Authors:** Raquel Moya-Costa, Gloria Cuenca-Bescós, Blanca Bauluz

**Affiliations:** Aragosaurus-IUCA, Department of Earth Sciences, University of Zaragoza, Zaragoza, Spain; Institut Català de Paleoecologia Humana i Evolució Social (IPHES), SPAIN

## Abstract

We have developed a protocol for reconstructing 3D models of the skulls of extinct species of small mammals. For the first time, the reconstruction uses fragments of fossils from a mixture of different specimens and from related extant species. We use free software and commercial computers to make the process reproducible and usable for the scientific community. We present a semi-quantitative protocol to face the problem of making 3D reconstructions of fossil species that are incomplete in the fossil record and/or represented by a mixture of different individuals, as usually occurs with small vertebrates. Therefore this approach is useful when no complete skull is available. The protocol combines the use of microCT scan technology with a subsequent computer treatment using different software tools for 3D reconstruction from microCT and 3D design and printing (e.g. Fiji, SPIERS, Meshlab, Meshmixer) in a defined order. This kind of free and relatively simple software, plus the detailed description, makes this protocol practicable for researchers who do not necessarily have great deal of experience in working with 3D. As an example, we have performed virtual reconstructions of the skulls of two species of insectivore small mammals (Eulipotyphla): *Beremendia fissidens* and *Dolinasorex glyphodon*. The resulting skulls, plus models of the extant shrews *Blarina brevicauda*, *Neomys fodiens*, *Crocidura russula* and *Sorex coronatus*, make it possible to compare characteristics that can only be observed by means of microCT 3D reconstructions, and given the characteristics of the material, using this protocol. Among the characters we can compare are the position of the mandibles, the spatial relations among all the teeth, the shape of the snout and, in general, all parameters related with the anatomy of the rostrum. Moreover, these reconstructions can be used in different types of context: for anatomical purposes, especially to see internal features or characteristics at whole-skull scale, for bioengineering, animation, or other techniques that need a digital model.

## Introduction

### 3D reconstructions from fossils

In recent years there has been an increase in the use of virtual palaeontology, in particular in three-dimensional reconstructions based on tomographies obtained from the techniques of computed axial tomography (CAT) or micro-computed tomography (microCT), in palaeontological studies [1–3]. This has occurred due to the high quantity and variety of applications derived from this type of reconstruction, including the possibility of extracting the fossil data from the rock that contains the fossil, sharing this information, studying the interior cavities, structures and materials of the fossil, characterizing fossils in 3D, retrodeforming fossils that have undergone plastic deformation, digitally reconstructing their palaeobiology, testing the movements and insertions of bones, and performing functional analyses using reverse engineering tools such as finite element analysis or hydrodynamic studies (i.e. [1–10]).

These techniques are especially useful for studying microfossils whose small size makes traditional techniques difficult to apply [6,11–13]. This is firstly because the microCT and subsequent reconstruction allow reproductions of the material to be handled without being touched, making it possible to clean them virtually, see them better, cut them and compare them. Second, it is because work with 3D virtual models can be at any scale, so the advantages in handling and cleaning are equal for all sizes of fossils, as long as all relevant specimen features are above the lower resolution threshold.

### The fossil record

The preservation of fossils plays an important role in the techniques needed to reconstruct them so as to work with them in 3D. Reconstructions using virtual palaeontology are based preferentially on complete fossils or on parts associated with the same individual that can be put together. When fossils are incomplete, the lacking parts can be inferred quantitatively using landmarks and semilandmarks. This is a common approach in anthropology and palaeontology (e.g. [14–17]), but this method needs one complete reference specimen. Sometimes it is not possible to obtain complete elements of some animals at certain palaeontological or archaeological sites. This is especially frequent in small-mammal accumulations in caves due to the entrance of allochthonous materials [18]. In these cases the small-mammal remains are disarticulated and more or less fragmented. When other animals such as birds of prey, carnivores and humans produce the accumulation, moreover, this can cause more severe fragmentation and further loss of bone elements. The remains are thus disarticulated and fragmented, making it difficult to assign different fragments to any particular individual. In addition, in such sites the remains of dozens of individuals may be mixed up amongst one another. They may also occur within the sediment, making it necessary to clean them by the method of washing and sieving [19,20] prior to the study. This introduces even more fragmentation, making it even riskier to assign the remains to the same individual.

There are particular instances where almost complete skulls and long bones are present. Unfortunately, the most complete fossils are usually found protected by concrescences that are difficult to remove without affecting the fossil due to the millimetre-scale size and fragility of the objects.

### Why Soricidae?

All small mammals and many small vertebrates present the problem of a fragmentary fossil record and thus the difficulty of reconstructing the species for palaeobiological study. Here we have chosen two soricids as an example to illustrate one way of attenuate this problem.

The family Soricidae (Eulipotyphla, Mammalia) comprises the “real shrews”. They appear in the fossil record for the first time during the Oligocene, roughly 35 Ma [21].

They are insectivorous small mammals that usually inhabit humid environments. In addition, some extant species have venomous saliva [22–24]. In general, they have a high metabolism, tiny eyes, and their most important sense is of smell, which influences the shape of their skull: this is characterized by big mandibles with teeth adapted to cutting soft food as well as chitinous shells, an absent zygomatic arch, and a strong and robust maxilla and highly sutured nasals that connect with the long, cartilaginous nose [25].

The teeth have one special adaptation in the subfamily Soricinae: they have iron oxides or hydroxides among the apatite crystals of the enamel [26–28].

Another special biological process that occurs in shrews is Dehnel’s phenomenon. This consists of the reabsorption and regrowth of the posterior part of the skull during the life of the shrew in accordance with the season [29]. This biological process also contributes to the difficulty in preserving the skull, as the bone of the braincase is especially weak, by contrast with the snout.

Many different genera of shrew are found in the fossil record, ranging from the Oligocene [21] to the present, most of them extinct today.

Two examples of extinct shrews are *Beremendia fissidens* and *Dolinasorex glyphodon*. These two species are found in the Early Pleistocene sites of the Sierra de Atapuerca. *B*. *fissidens* first appears in the Pliocene of China, and survives in Eurasia until the end of the Early Pleistocene [30,31]. By contrast, *Dolinasorex* is found exclusively in the Early Pleistocene layers of the Gran Dolina site in Atapuerca, in the north of Spain [32]. Both species disappear at the end of the Early Pleistocene, are larger in size than the other genera of Soricidae in the Early Pleistocene, have red teeth, have a divided mandibular condyle, have robust mandibles and molars, and possess a possible envenomation apparatus consisting of a groove present in the lower incisors [30–35]. The differences between them consist in the larger size of *D*. *glyphodon*, the different proportions of features such as the wider coronoid process of *D*. *glyphodon*, and the fact that the latter appears in Gran Dolina whereas *B*. *fissidens* appears in Sima del Elefante, an even older site at Atapuerca [32,34]. Given their similarities, *D*. *glyphodon* could be ethologically similar to *B*. *fissidens* [32], which inhabits temperate-humid environments [34] and–like all shrews–has a diet certainly based on earthworm and arthropods, but which may have also specialised in hunting bigger prey such as small vertebrates [32,34] or by contrast specialised in storing and chewing hard invertebrates such as beetles or snails in relation with a possible fossorial lifestyle [35].

As small mammals are used to infer the palaeoenvironment of the levels where they appear, it is important to know about the lifestyle of *B*. *fissidens* and *D*. *glyphodon* to gain a better understanding of the evolution of the environment at the sites of Atapuerca,

### Objective

Here we propose a protocol combining different methods in order to attenuate the problem of the fragmentary fossil record of small mammals, specifically using the example of two shrews. The goal is to develop a way of reconstructing the most complete virtual 3D skull from fragments of fossils from different individuals.

We present an option for making semi-quantitative reconstructions using free software and commercial computers, and develop a detailed protocol for using them to this end. The aim is to make this process accessible for people with different interests, working in different fields related with small fossil vertebrates, and who do not need a deep background in this kind of work. The resulting models should be both as accurate as possible and useful for different kinds of study, allowing measurements in different directions and between elements positioned in their anatomical position, as well as the study of the interior of the skulls, among other applications. It can be also useful for activities of dissemination such as museum exhibits.

## Materials and methods

### Material

Fragments of the skull of *B*. *fissidens* and *D*. *glyphodon* were used. Selection was based on the best-preserved and most complete fossils. When a particular part was lacking, another fossil that had the part in question was selected.

**Fig 1:**
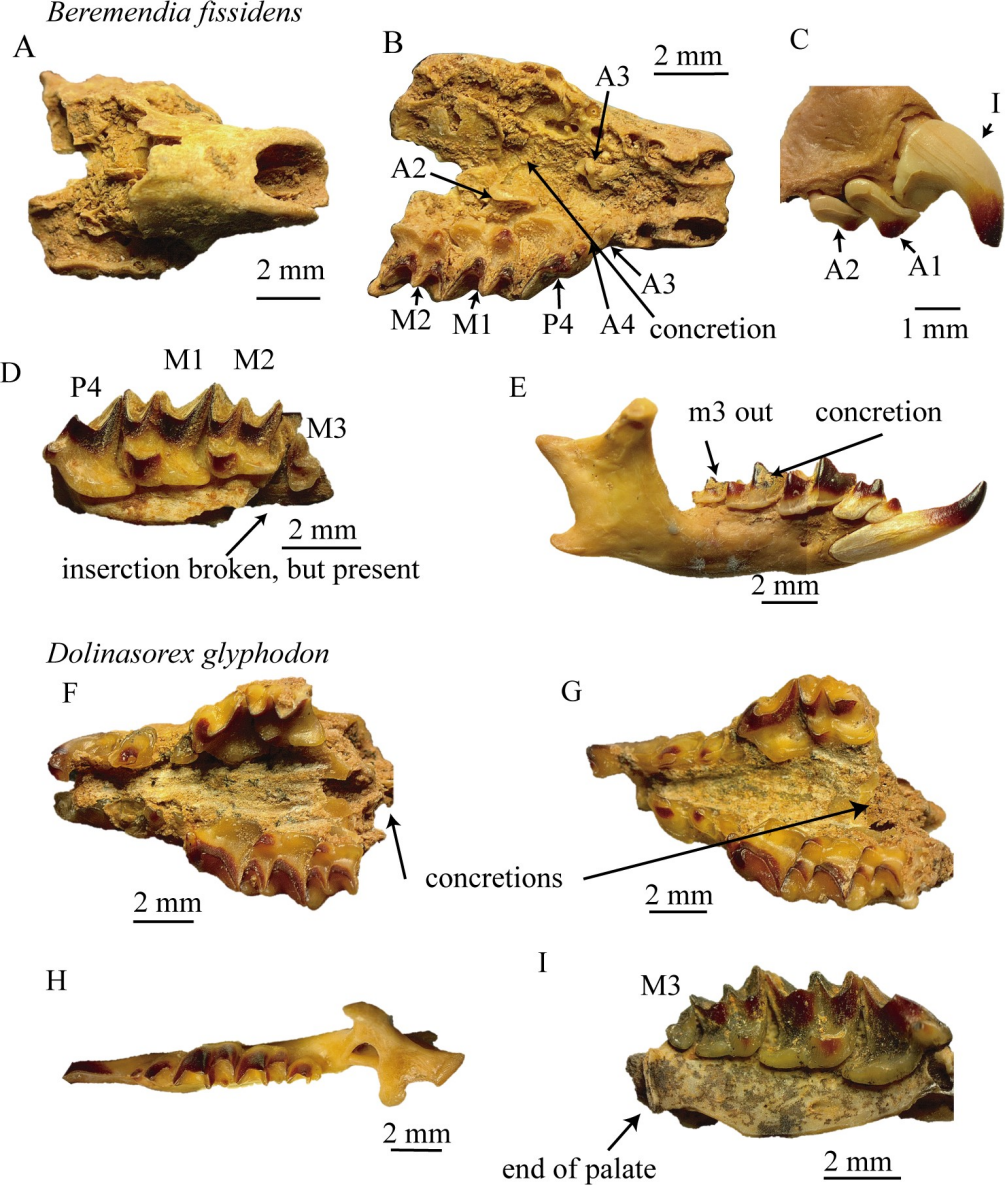
Fossil material. Fossils used to make the 3D-reconstructions. A-E *Beremendia fissidens*. A and B, upper and lower view of MPZ 2018/467, C fragment of anterior part of maxilla MPZ 2018/468, D fragment of maxilla with M3 MPZ 2005–491, E labial view of complete mandible with teeth MPZ 2018/469. F-I *Dolinasorex glyphodon*, F lower view of one concreted and incomplete skull MPZ 2010–512, G lower view of another concreted and incomplete skull MPZ 2010–201, H upper view of a complete mandible MPZ 2010–579, I lower view of a fragment of maxilla that preserves the posterior part and the M3 MPZ 2010–27.

The fossils selected are described and shown in Fig 1.

In addition, we selected a complete *Blarina brevicauda* skull with mandibles in order to have a complete reference from a closely related species, especially in the light of the fact that the braincases of fossil species are not preserved. The easiest way of getting a complete skull from a species related with the species of interest is to use an extant species. We have chosen *B*. *brevicauda* because it is also a soricine. Some authors include both *Blarina* and *Beremendia* in the same tribe, Blarinini Stirton 1930 [36], but the main reason for our choice is that works on the palaeobiology or palaeoecology of *Beremendia* and *Dolinasorex* [32–35] always compare these fossil shrews with *Blarina* on account of their similarities, such as the short and robust snout and mandibles, the dark pigmented teeth, the divided mandibular condyle and the venomous saliva. These characteristics may be even more important than phylogeny in selecting the complete skull for carrying out the reconstruction, as some studies show [37].

In addition *Blarina* is a large shrew, as the species that we want to reconstruct [34,35]. The difference in size is important because a large difference in size can produce variation due to allometry [38–40]. This phenomenon could be attenuated using as complete skull a model predicted from allometric regression, but this would require a previous large scale study, which is not always possible due to paucity of material.

The *D*. *glyphodon* material comes from the Gran Dolina site (TD); the *B*. *fissidens* material comes from the site of Sima del Elefante (TE); all the fossil material is stored in the Museo de Ciencias Naturales de la Universidad de Zaragoza [41].

#### B. fissidens

- Concreted anterior part of the skull with part of the maxilla and nasal bones and the left A3, A4, P4, M1 and M2 in their alveolus. The left A2 and right A3 are also present, but isolated and stuck to the palate. The part where M3 is inserted is missing in the maxilla. It is from level TE9c of Sima del Elefante. MPZ 2018/467.
- Fragment of maxilla with the right I, A1 and A2, from level TE9c. MPZ 2018/468.
- Fragment of maxilla with the left P4, M1, M2 and M3. The maxilla is broken in the part where M3 is inserted, but all the fragments are together. It is from level TE9a. MPZ 2005–491.
- Right mandible. Complete with all the teeth and partially concreted. It is from level TE9c. MPZ 2018/469.

#### D. glyphodon

- Complete right mandible with all the teeth, from level TD6. MPZ 2010–579.
- Fragment of maxilla with right P4, M1, M2 and M3 and the posterior part of the palate preserved, from level TD5E. MPZ 2010–27.
- Concreted maxilla with the left I, A1, A2, A3, P4 and M1 and the right A1, A2, A3, P4, M1 and M2. The insertions of M3 are preserved. Nasals are not preserved. It is from level TD5BE. MPZ 2010–512.
- Maxilla and nasals concreted. With the left I, A1, A2, A3, P4 and M1 and right A2, A3, P4, m1 and M2. It is from level TD5E. MPZ 2010–201.

#### Extant species

In addition, remains of extant shrews were used to complete the reconstruction of the skulls and to compare the final models of different species. The reason to choose them is that they are complete. In this work they come from owl pellets because of ease of access, as this kind of remains is available in museum collections:

- One skull and the two hemimandibles corresponding to the same individual of *B*. *brevicauda*, from North America. Material purchased already prepared. Universidad de Zaragoza.
- One skull and one hemimandible of *Neomys fodiens*, obtained from owl pellets in the Pyrenees. Material from the Instituto Pirenaico de Ecología (IPE)
- One skull and one hemimandible of *Sorex coronatus*, obtained from owl pellets in the Pyrenees. Material from the Instituto Pirenaico de Ecología (IPE)
- One skull and one hemimandible of *Crocidura russula*, obtained from owl pellets in the Pyrenees. Material from the Instituto Pirenaico de Ecología (IPE).

### Methods

The material that comes from recent vertebrates was borrowed from the Instituto Pirenaico de Ecología, which recovered them from owl pellets. We did not perform the sampling. Animals were not sacrificed. The skull of *Blarina brevicauda* was purchased.

The fossils of the samples studied come from field archaeological studies under the direction of Juan Luis Arsuaga, Eudald Carbonell and José María Bermúdez de Castro, who have permission from Junta de Castilla y León.

#### Recovery of the samples

The fossils in this work are from the archaeological sites of Sierra de Atapuerca (Burgos, Spain). Specifically, they are from the cave sites of Gran Dolina and Sima del Elefante (Burgos, Spain) and were collected in different excavation campaigns from 1996 to 2015. The microfossils were recovered using the method of washing and sieving sediments [42]. Each sample corresponds to a volume of sediment of 1m x 1m x 10cm and is labelled with the exact level and spatial position in the site. In the course of each campaign the sediment was washed and sieved using sieve towers with decreasing meshes (5, 2 and 0.5 mm); water was extracted by water-pump from the River Arlanzón in order to remove the clay that covers the fossils. The resulting concentrate is formed of small clasts and fossils of small vertebrates or fragments of large vertebrates. Finally, the identifiable fossils were picked out and separated with soft forceps. We selected the fragments of shrews detailed in the “Material” section from this mixture of fossils collected in the field and from material stored in the Museo de Ciencias Naturales de la Universidad de Zaragoza (MCNUZ) [38] because of previous studies that had also selected the material from the concentrates.

Having described the selection and collection of the material, we now explain the protocol designed and followed in the reconstruction of both skulls:

#### Digitalization

Prior to digitalization, images of all the elements were taken in the laboratory with an Olympus SZ61 trinocular microscope and an Olympus Soft Imaging Solutions LC20 camera, and measured with the software TpsDig v.2.17 [43].

The fossils and present-day items were scanned in the Centro Nacional para la Investigación de la Evolución Humana (CENIEH, Burgos) using a Phoenix v/tome/x s by GE Measurement & Control. The technique used was the microCT scan.

The voxel size was isometric, with the same value in X, Y and Z. It was 9.49978 μm in all samples except in *B*. *fissidens*, where it was 7.99958 μm, and MPZ 2010–201, where it was 9.50016 μm. The pixel size in X and Y was always 0.2 μm. The voltage used was 100 kV, and the current 140 ρA.

Beforehand, the pieces were set up for scanning. Some of them were put inside small plastic containers as centrifuge tubes and wrapped in plastic (HDPE) to avoid interference between the container and the piece, and make sure that the piece would not move inside the container during scanning, since the scanning involves the rotation of the piece. The biggest samples, such as the *B*. *brevicauda* skull, were fastened inside an expanded polystyrene container that had previously been adapted to the skull morphology. Some of the samples had been stored in a museum for years, glued with a reusable putty-like adhesive that had hardened during this time.

The computer that was used to make the reconstructions was a commercial laptop, so the process of creation is adapted to its characteristics: Windows 10 system with processor Intel Core i7-7700HQ CPU 2.80GHz, 16 GB RAM memory, 64-bit system, and the graphic card is NVIDIA GeForce GTX 1050 Ti, with 12 GB of memory.

The data were exported as TIFF images. They were treated with the software Fiji (ImageJ) [44–46] to optimize the size of the files. The quality was reduced from 16 bits to 8 bits, the work area was cut and adapted to the elements, and then the images without bony elements were deleted. The images were also rescaled.

With the software XnView v.2.38 [47], the TIFF format was converted to bmp because TIFF is not accepted in the software here used for the 3D reconstruction.

#### Creation of the 3D models

The 3D models of each piece were created using the free software SPIERS [48, 49], with the application SPIERSEdit v.2.20.

No downsampling was applied to the models of fossils, but a downsampling of four was applied to the skulls in the recent material in order to reduce the size of the files and facilitate work with a commercial laptop. The reason we decided not to apply downsampling to the fossils, which are the objects of greatest interest, was that it reduces the resolution of the model, but we did apply it to the extant species, which are used for comparison [50].

To select the area of the images that had to be reconstructed, a different threshold was applied to each piece due to the varying range of greys obtained in the scans. The threshold was applied trying to exclude most of the external elements such as the container and protection of the pieces, the concretions, the hardened adhesive stuck to some of the pieces, and the glue that was reinforcing the pieces of *B*. *brevicauda*. As it was not possible to remove some of these elements completely (e.g. the concretions and putty-like adhesive) because their grey contrast coincided partially with that of the bone or enamel, we also tried to select a threshold that did not exclude the material under study.

After this, the Mask mode of SPIERSEdit was used, and different masks were used to separate elements of each piece. Both the teeth of the complete maxillae and those that belonged to incomplete maxillae that were needed for the reconstruction were separated.

If the external bones were broken, they were also separated in order to put them in their correct position later. Using masks, the external concretions and putty adhesive were avoided or marked. For the non-fossil material, the elements were not separated. Each masked element was converted into an object and exported to SPIERSview v.2.20. From SPIERSview the objects were exported into Virtual Anatomy eXtensible Markup Language/Standard Triangle Language (VAXML/STL). The parts unnecessary for the final model were not exported.

Each object was first importedinto MeshLab v2016.12 [51]. There we applied the filter “Invert Faces”. Then the objects were exported into PLY (Polygon) file format.

To continue with the treatment of the mesh, the PLY files were then imported into Meshmixer [52]. There we used the “Inspector” analysis tool and the “Auto Repair” option to remove the islands corresponding to the dirt on the model.

It was necessary to smooth the models using manual smoothing brushes. The smoothing affects the model to different degrees depending on the structure, so it is important to be careful in performing this step [50]. The “ShrinkSmooth” brush was used over the whole surface of the objects in order to delete the “step” effect produced by the reconstruction of the slices, which is a consequence of the method of digitalization and reconstruction and thus does not represent real structures. “RobustSmooth” was used if there was any irregularity associated with extra material such as concretion stuck to the pieces. If the imperfections were related to cracks or to missing material, they were treated using “BubbleSmooth”. The size and strength values of the brushes were low, adapted to the shape and size of the triangles so as not to distort the general shape of the pieces. The effects of the application of smoothing on different types of structures are explained in [50]. We show the effects produced on some elements in the figures in S1 Fig.

Finally, the number of triangles was reduced using the “Reduce” brush, which is a decimation filter. The strength and size of the brush was also adapted to the piece and the surface, being low in the teeth, especially in details such as crests and cusps, and higher in the bones, in the flatter surfaces. This was done with the “Wireframe” view to ensure that only the unnecessary triangles were deleted. It is important to be careful here in order not to lose structures [50].

#### Formation of the general models

As each of the separated elements of each fossil was saved in the same position, they were opened in the same file. Firstly, the broken parts of bones such as the insertions for m3 were reconstructed using the “Transform” tool to collocate the fragments in their correct position with respect to the main fragment. The teeth were also relocated in the cases where they had popped out from their alveolus, totally or partially.

Later, with all the elements selected at the same time, the “Transform” tool was used again to rotate the elements in order to orientate them in the same way, that is:

- The antero-posterior anatomical axis corresponds with the front-back direction of Meshmixer, or the X axis.
- The lateral anatomical axis corresponds with the right-left direction of Meshmixer, or the Y axis.
- The dorso-ventral anatomical axis corresponds with the top-bottom direction of Meshmixer, or the Z axis.

After this, the elements were scaled to form the final model. In *B*. *fissidens* the most complete maxilla was used as a reference, whereas in *Dolinasorex* it was one of the snouts without the complete nasals that was used. The other models were added to the file and scaled using the length of the teeth “repeated” in the models, i.e. the length of A2 and the width of M2 for the upper elements. For this scaling, the tools used were the “Measure” selection in “Analysis-Units and Dimensions” and then, after calculating the scale value, “Edit-Transform-Scale”.

For the lower dentition, as the teeth were not separated in the initial model, a different process was applied to correct the position of the m3, which was also rather detached from its alveolus. The m3 was selected in the model by combining the “Lasso” tool, where it was possible to select all the structures of the tooth, and the “Brush” selection tool. Then it was separated and transformed so as to be put in its position. The scale of the mandible with respect to the upper dentition was calculated by measuring the distances between the cusps of the lower teeth and making them equal to the distances between the points where they occlude in the upper dentition.

Due to the symmetry of vertebrates, only the elements of one side were selected, although the most important factor was the preservation of the elements rather than whether they were all from the same side. For this reason it was necessary to mirror the pieces [7,15], which means duplicating them with the opposite orientation with respect to a plane of symmetry. Here the plane used was the sagittal plane.

The first step was to select the better-preserved half of the maxilla and nasals. In *Beremendia* it was the left half that was selected, but in *D*. *glyphodon* the anterior part of the left side was broken whereas the posterior part of the right side was missing, so the *D*. *glyphodon* maxilla was mirrored by sectors. To undertake this process, the models were cut using the “Plane cut” editing tool. The cutting plane was located in the sagittal plane of *B*. *fissidens*, and in the case of *D*. *glyphodon* parallel to this plane at two different points to avoid the higher quantity of imperfections, and also perpendicular to the coronal plane in the position of the premolars. The type of cut used was the “Slice (Keep Both)” option. To separate the slices as different objects, it was necessary to use the “Separate Shells” edit option. The interesting parts were then mirrored with the “Mirror” edit option in the correct position, taking the real object as a guide. Afterwards, the real objects that we wanted to replace were deleted. With the elements that had not been cut exactly in the sagittal plane but parallel to it, after the mirroring and separation it was necessary to transform them to set the correct distance to the middle.

With other pieces such as the teeth or mandible, the mirror was applied with respect to the sagittal plane, bearing in mind the correct insertion of teeth in the alveolus and the occlusion of upper and lower teeth.

Once this was done, the whole model was scaled using the height of the mandible. This was 6.15 mm for *B*. *fissidens*, a small shrew whose size lies within the range of measurements for Sima del Elefante and is rather smaller than the mean for Europe, 6.28mm [34]. The measurement for *D*. *glyphodon* was 7.16 mm, which coincides exactly with the mean from level TD6 of Gran Dolina [32].

#### Reconstruction using a complete skull of a related species

At this point, the models are the most complete skulls that can be obtained from real fossils of these species. However, these models lack the posterior part of the skull, the whole braincase, including the temporomandibular joint. In order to gain an idea of what this part might be like, the next step was to attach the posterior part of *B*. *brevicauda* to the models of the fossils. In the “Materials” section, we have explained why we selected a skull of this species. To this end, the model of the *B*. *brevicauda* skull was imported into the Meshmixer file of the fossil reconstruction.

The model of the skull of *B*. *brevicauda* was scaled using the length of the maxilla and superimposed upon the models of *B*. *fissidens* and *D*. *glyphodon*.

In *B*. *fissidens*, when the length was scaled, the width of the maxilla and height of the snout did not coincide, so it was necessary to add a non-uniform scale of 110% to the Y and Z axes. In the case of *D*. *glyphodon*, by contrast, this latter transformation was not necessary.

After making the maxilla coincide in size and shape, the only part that could be inferred, at least in position, was the glenoid fossa. This is because one of the parts of the temporomandibular articulation, namely the condyles of the mandible, is oriented in the position of occlusion. In both cases, the glenoid fossa had to be extended outwards in a lateral direction, because in the fossil species the mandibles are wider in form and position than in *B*. *brevicauda*. As the transformation is not uniform, the transformation was here carried out using the “Drag” brush, dragging the whole of the articulation and the surrounding part to make it coincide with the mandible. In *D*. *glyphodon* it was also dragged in an anterior direction because the mandible is proportionally shorter in the posterior part.

**Fig 2:**
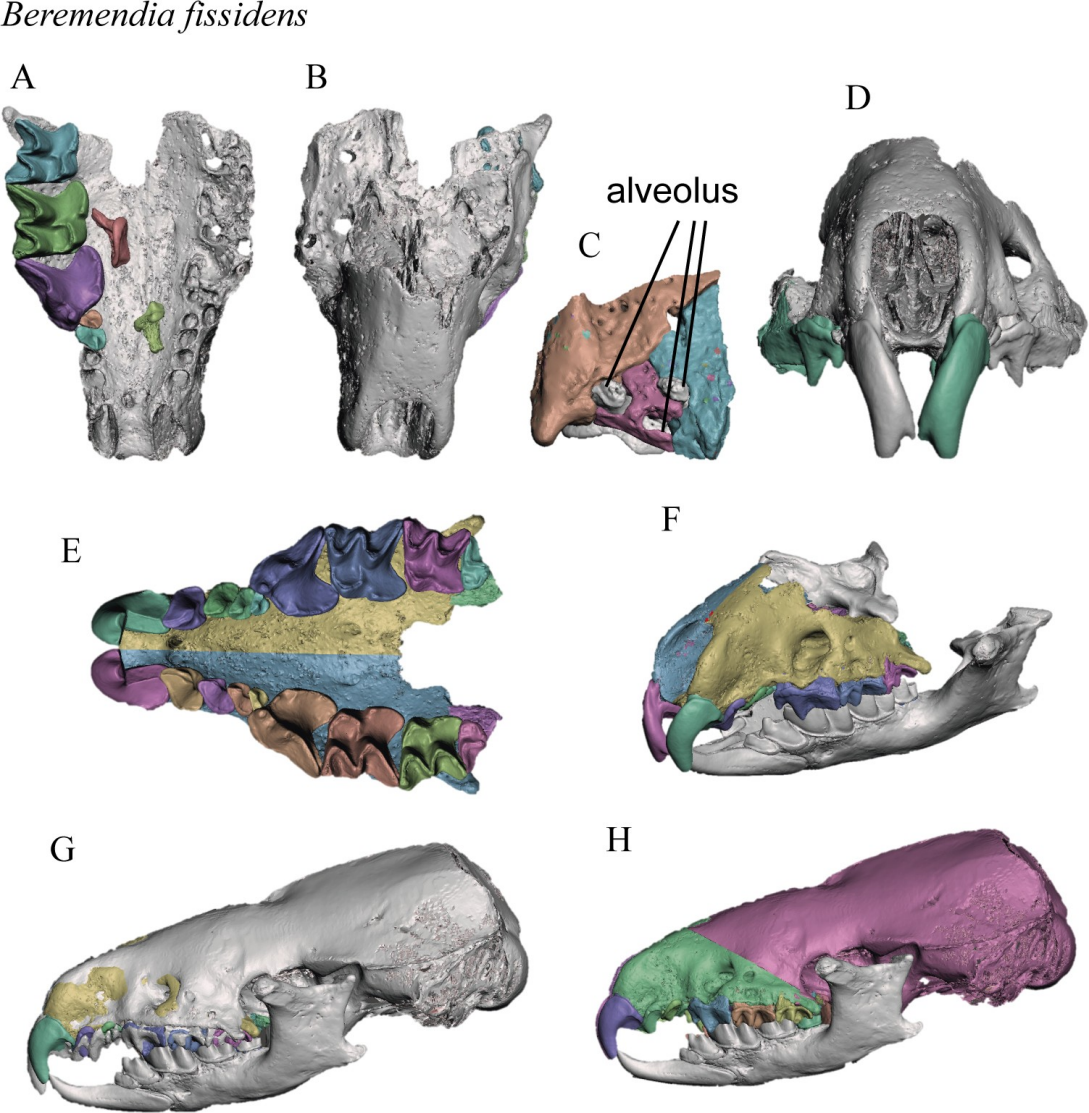
Reconstruction of *Beremendia fissidens*. A. Separation of the elements of the main fossil, lower view. B. Upper view of the maxilla used. C. M3 insertion part reconstructed. D. Frontal view of the original maxilla with teeth mirrored. E. Lower view of the reconstructed skull. F. Oblique view of the skull with the mandibles mirrored and collocated. G. Superimposition of the skull of *B*. *brevicauda*. H. Final reconstruction with the adapted skull of *B*. *brevicauda*. Different colours indicate different 3D objects.

**Fig 3:**
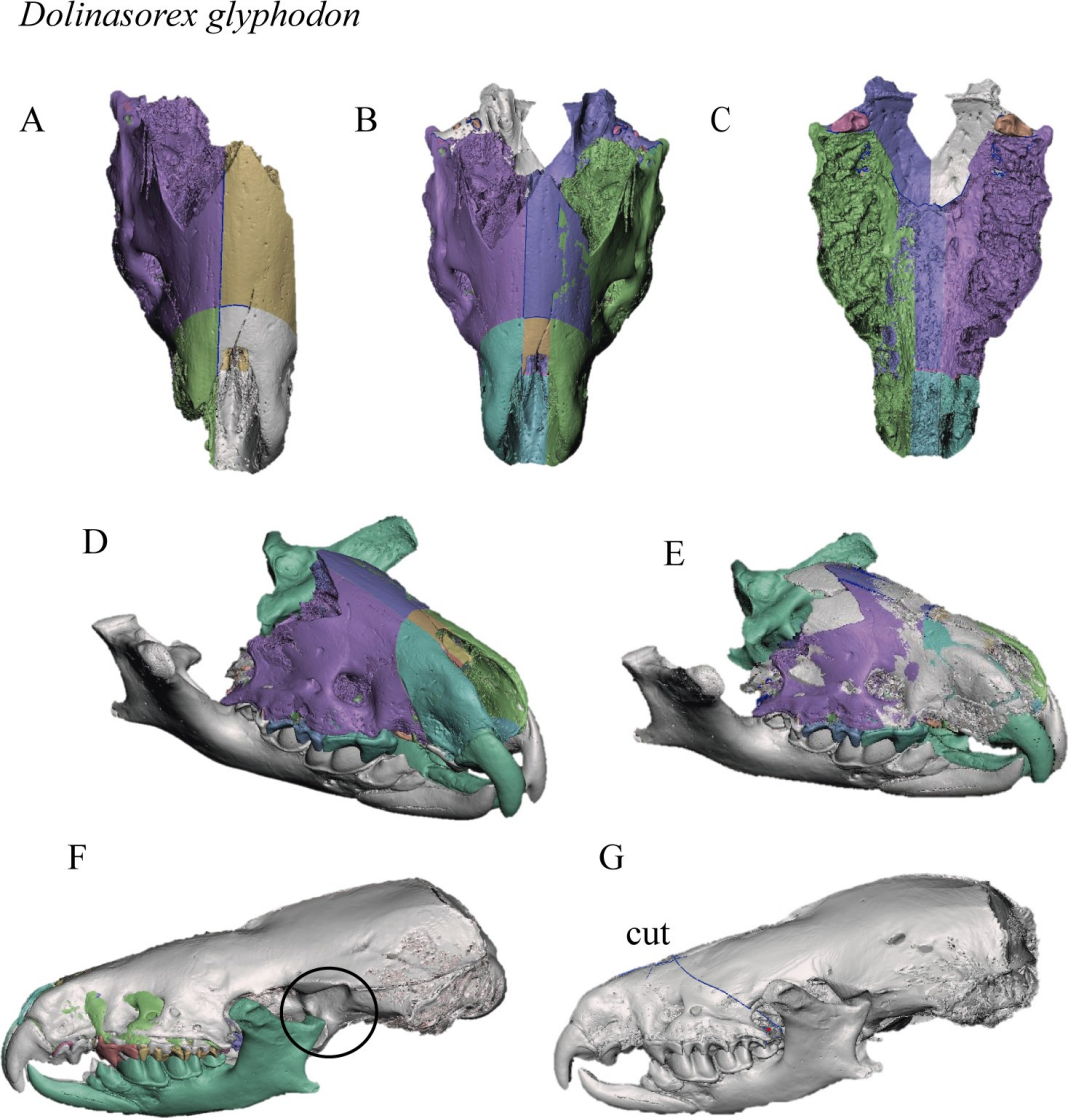
Steps in the division and reconstruction of *Dolinasorex glyphodon*. A. Reconstructed part of one skull, divided into the sectors that were subsequently mirrored. B-C. Upper and lower views of the reconstructed snout. D. Reconstruction with the snout and mandibles. E. Superimposition of the snout with complete nasals. F. Superimposition of the skull of *B*. *brevicauda*. The part with the worst adjustment is marked. G. Final reconstruction with the adapted skull of *B*. *brevicauda*. Different colours indicate different 3D objects.

The process is shown in Figs 2 and 3.

The reconstructions of the extant shrews were performed in a similar way, but mirroring only the mandible needed and without using an adapted *B*. *brevicauda* skull.

By this point we have obtained the two reconstructions that were the objective of the work. We also have four models of extant shrews to compare them with. The following section seeks to show some of the possibilities for working with the 3D models obtained from fragments, specifically in the study of these soricids.

**Fig 4:**
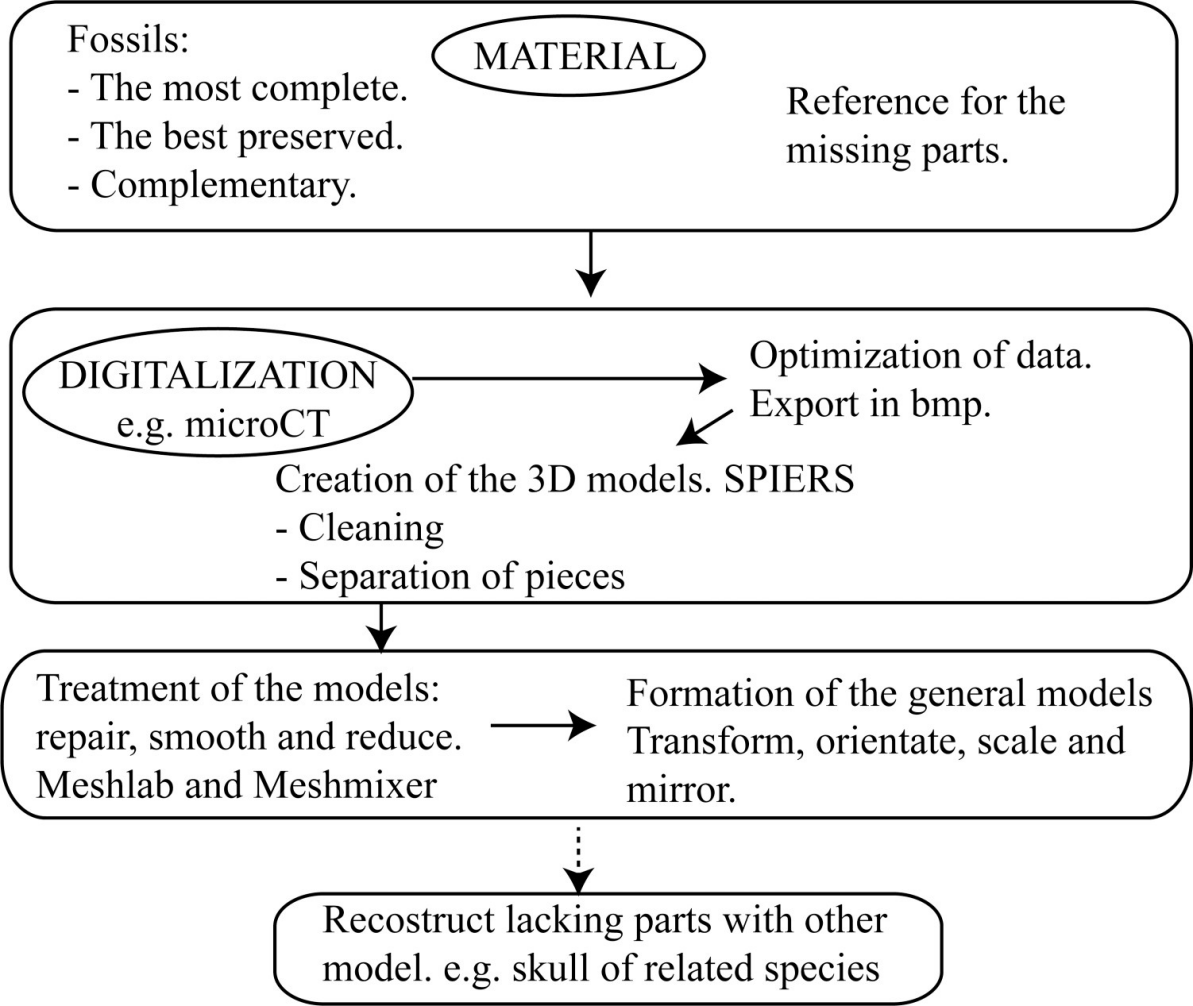
Steps. Flow chart that summarizes the main steps of the protocol to make a reconstruction.

The entire protocol to make the reconstructions is summarized in Fig 4.

#### Example of an application of the reconstructions: Measurements

**Fig 5:**
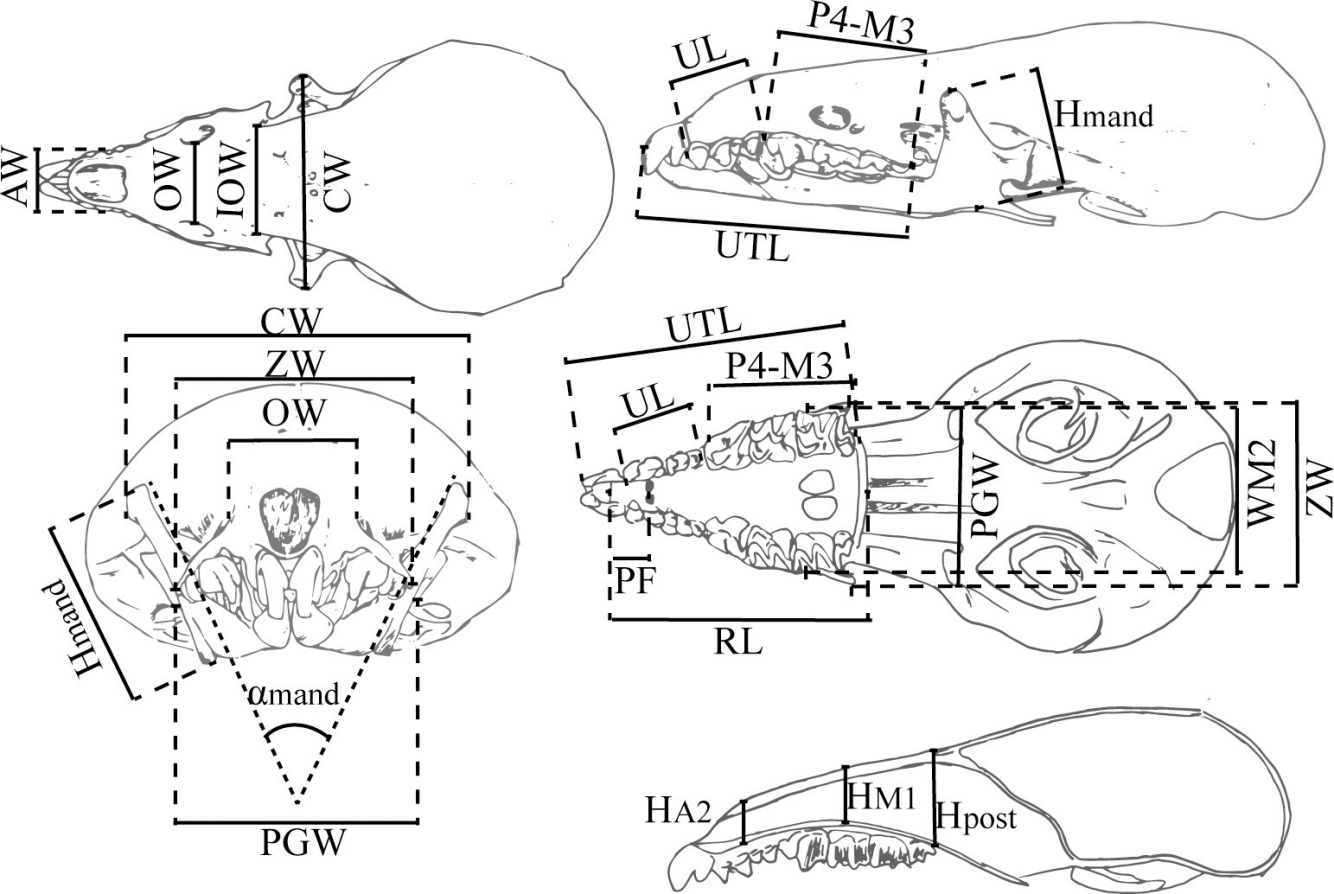
Measurements taken in this study for each species. Measurements taken in this study. Some of them are modified from [53,54].

Using the reconstructions, we took measurements of the anterior part of the skulls in order to compare the different species and characterise the anterior part of the skull of shrews. These measurements were taken with the “Analysis-Dimensions” tool in Meshmixer, except for the angle between the mandibles, which was measured in the frontal projection using TpsDig v.2.17 [43]. The measurements are modifications of those used in [53,54], which are shown in Fig 5. They are the rostral length (RL), distance from the palatine foramen to the anterior end (PF), length of the upper dental series (UTL), length of the unicuspid series (UL), length from P4 to M3 (P4-M3), infraorbital width (OW), interorbital width (IOW), anterior width in A2 (AW), width of M2 (WM2), zygomatic width (ZW), height of the snout at M1 (HM1), height of the snout in the posterior part of the palate (Hpost), height of the snout at A2 (HA2), coronoid separation (CW), postglenoid width, direct or inferred from the mandibular condyles (PGW), height of the coronoid process of the mandible (Hmand), and the angle formed by the two mandibles (αmand). Note that the measurements were selected to measure only the parts obtained with fossils, not the parts reconstructed with the *B*. *brevicauda* skull.

In addition, in order to make the measurements comparable among the different species, each one was divided by the rostral length of each reconstruction.

## Results

### Models

Six models of shrew skulls were obtained: two skulls from extinct species of shrews, one from *B*. *fissidens* and the other from *D*. *glyphodon*; and four from extant species: *B*. *brevicauda*, *N*. *fodiens*, *S*. *coronatus* and *C*. *russula*.

**Fig 6:**
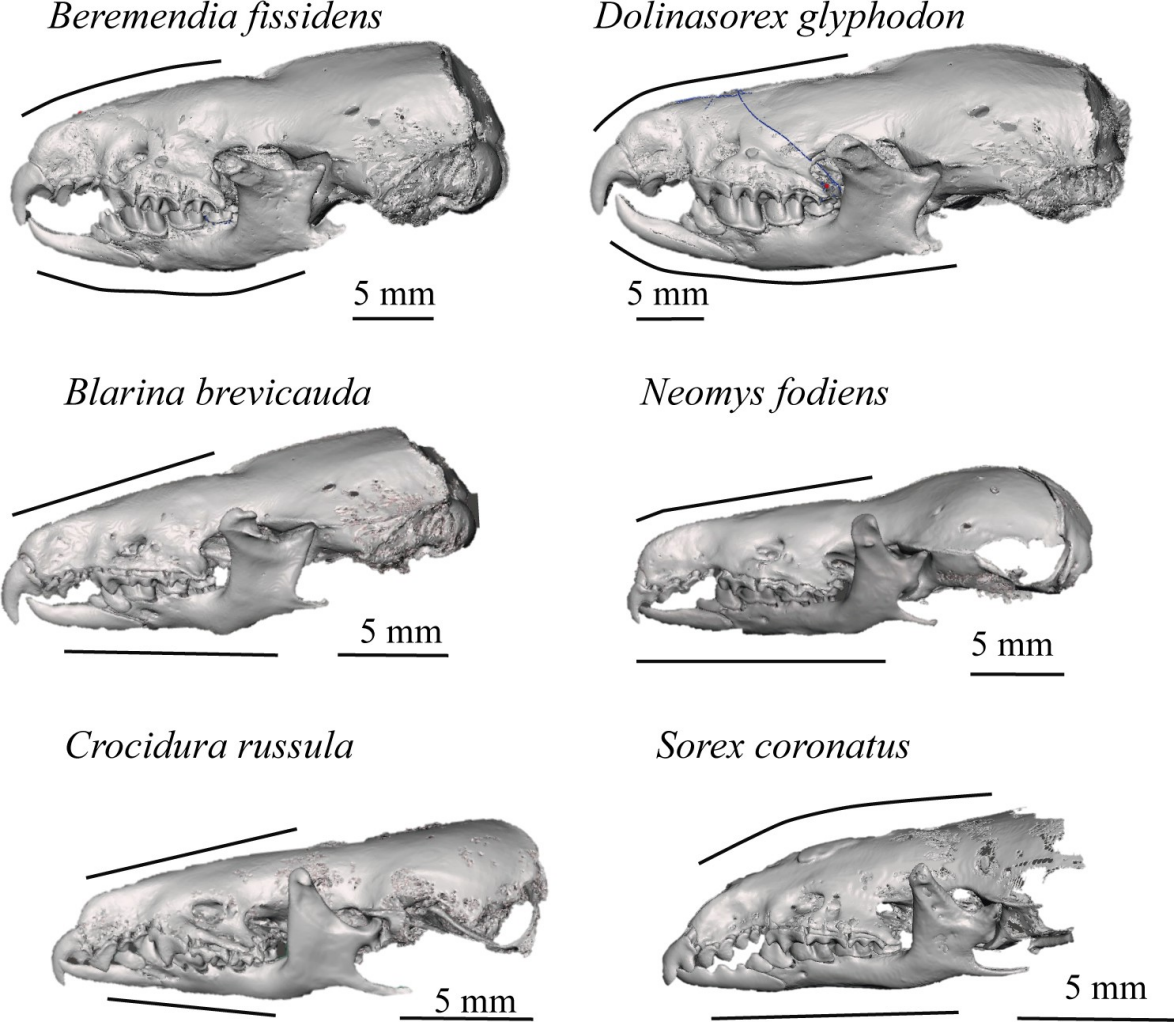
Lateral view. Lateral view of the skulls reconstructed.

**Fig 7:**
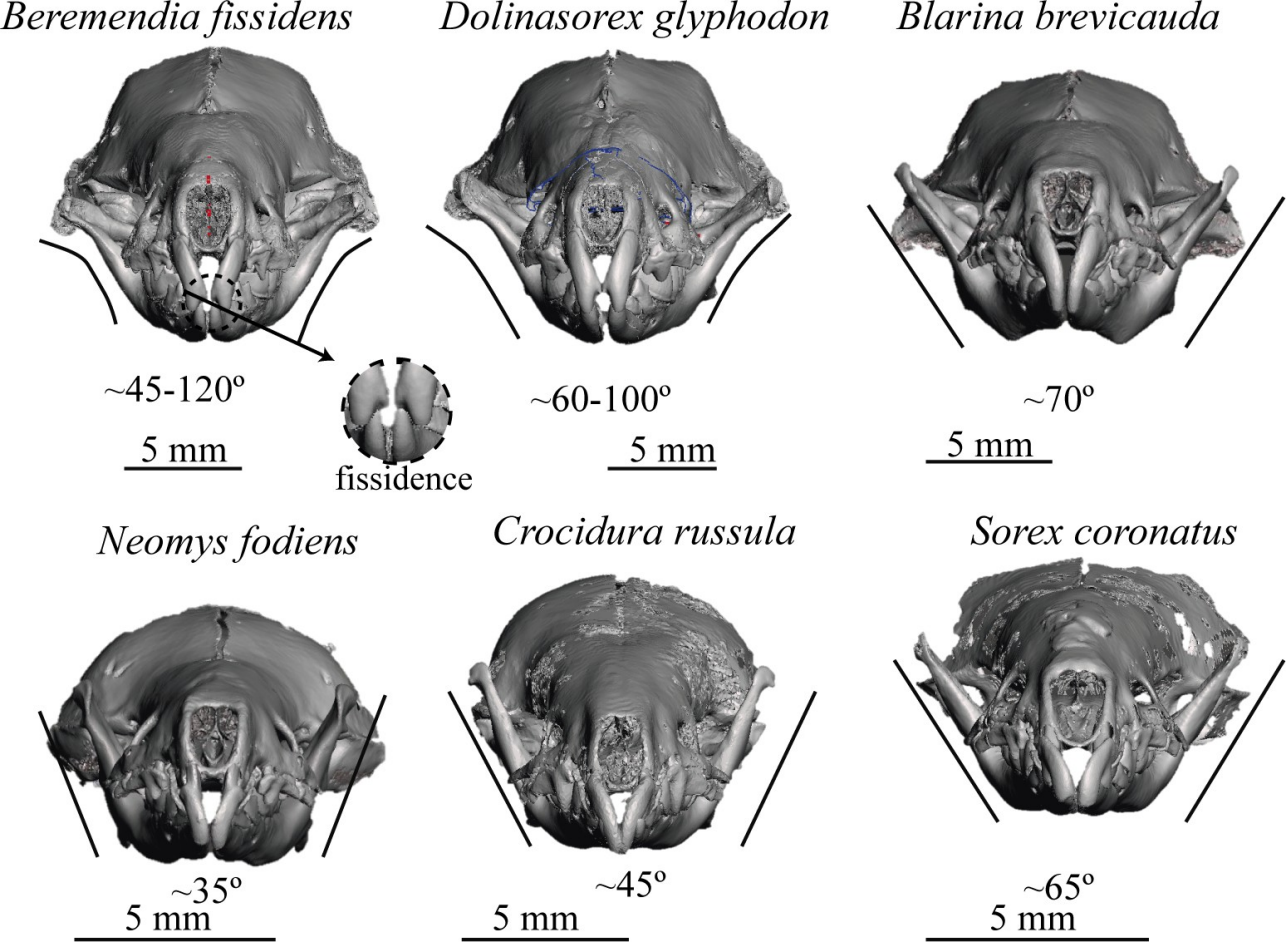
Frontal view. Frontal view of the skulls reconstructed.

**Fig 8:**
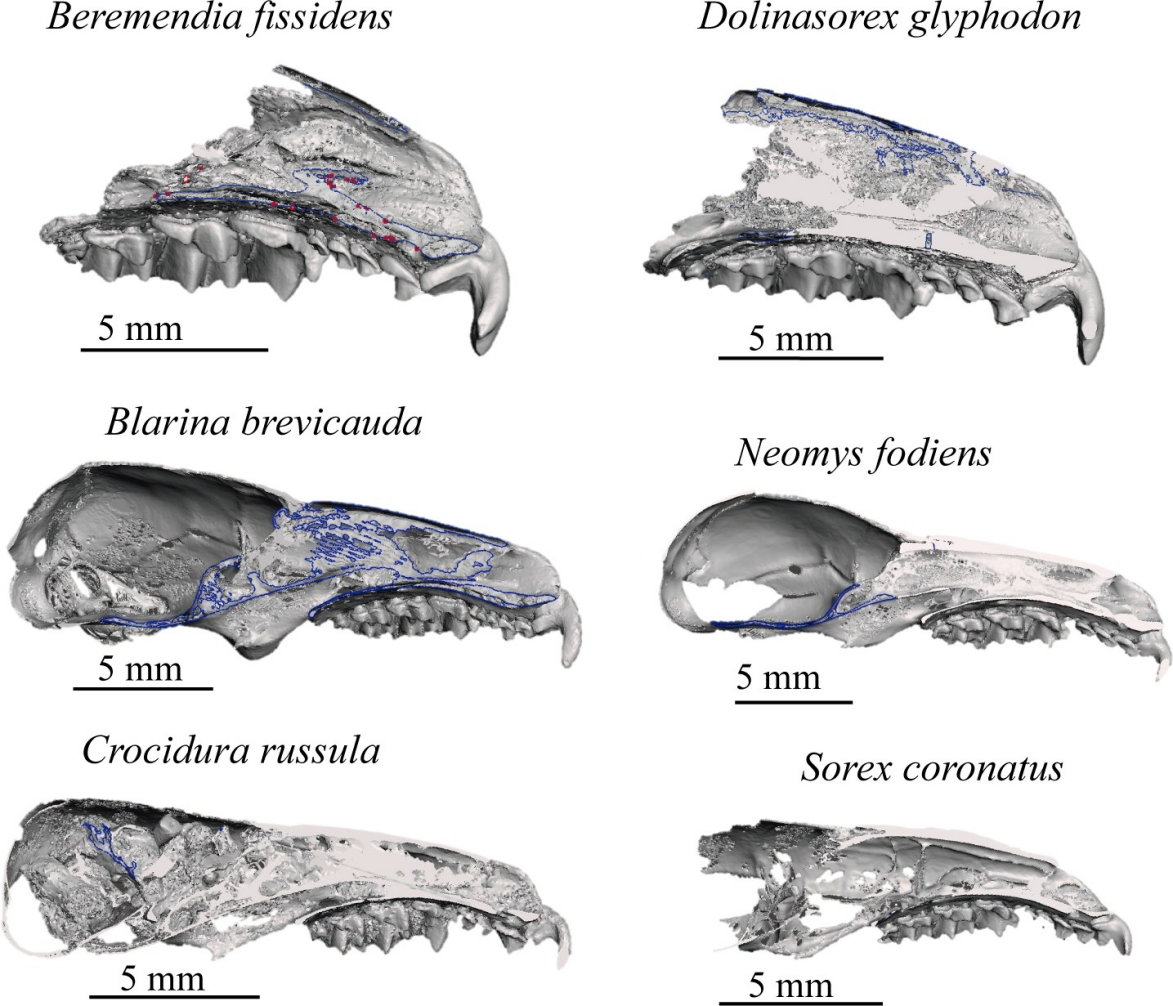
Sagittal cut. Sagittal cut of the skulls reconstructed.

**Fig 9:**
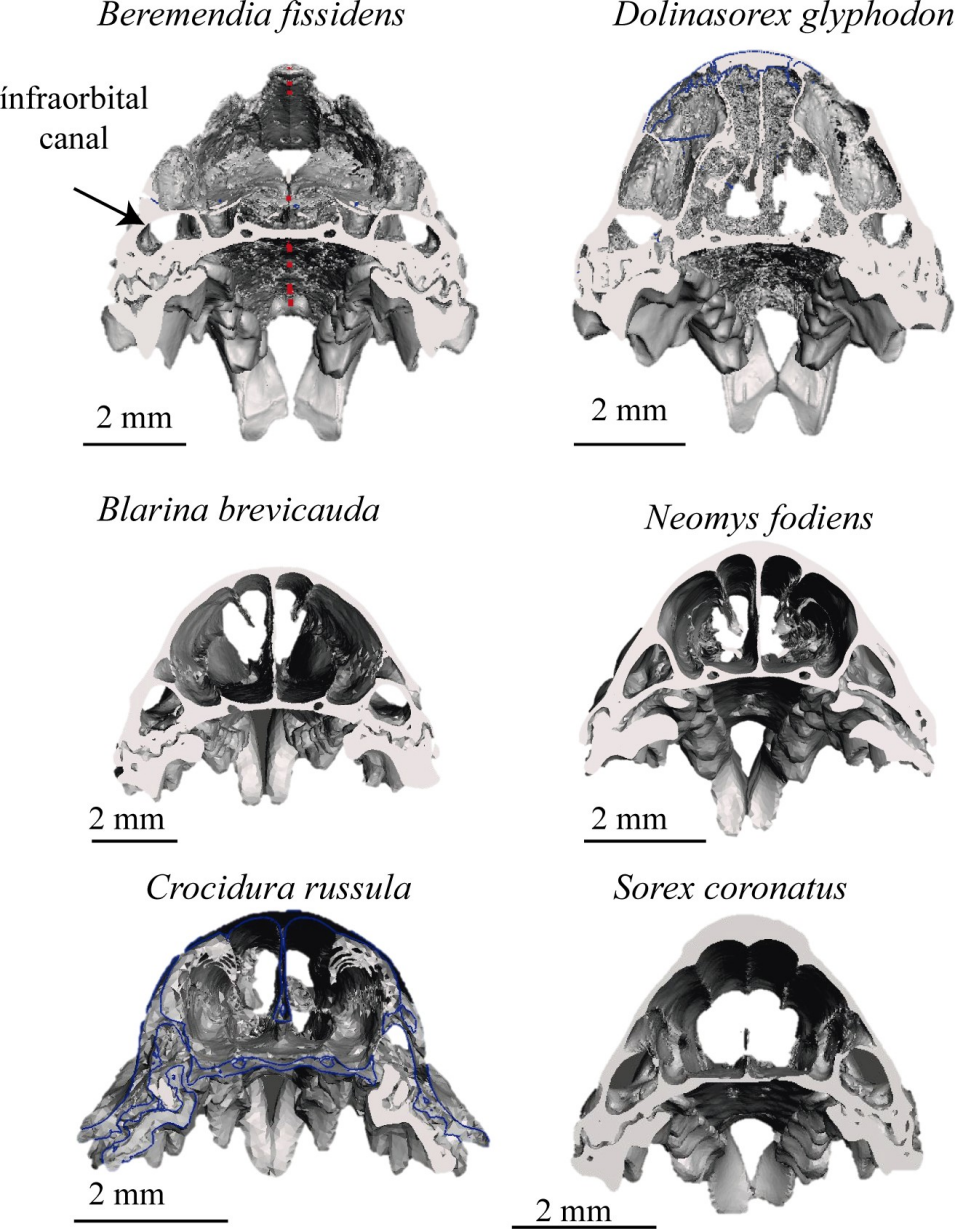
Coronal cut at P4. Coronal cut at P4 of the skulls reconstructed.

These models allow the reconstruction to be rotated and observed from whatever point we wish; they enable us to see the occlusion of teeth, view or hide the different elements that make up the model, such as the adapted skull, mandibles or teeth, and even cut through the models to see the interior of bones, teeth and skull. The lateral views of the models are shown in Fig 6 and the frontal views in Fig 7. In Figs 8 and 9 the sagittal and coronal cuts are shown. The models of the reconstructed fossil species are shown in S1 and S2 Files.

We have maintained the division between the adapted skull of *Blarina* and the mandibles and snout reconstructed directly from fragments of *Beremendia* and *Dolinasorex* in order not to mix them up, although the complete model is useful too.

*B*. *fissidens* is the species with the proportionally shortest and most rounded skull, whereas *D*. *glyphodon* has a longer snout but a robust and generally bigger skull, as [32] pointed out.

Here we describe some of the characteristics that can be compared using the reconstructions:

*B*. *fissidens* and *Sorex* have curved upper incisors, whereas in the other species the frontal shape (Fig 7) is more vertical and straight. In addition, in *B*. *fissidens* and *D*. *glyphodon* the incisors have medial tines. These are wider in *B*. *fissidens*. This gives them the “fissidence”.

The upper teeth with the biggest roots are the incisors, followed by the premolars. The incisor root is relatively bigger in *N*. *fodiens*, *B*. *brevicauda*, *B*. *fissidens* and *D*. *glyphodon* than in *S*. *coronatus* or *C*. *russula*. It is bigger than the crown in the former, and smaller in the latter.

In the coronal sections (Fig 9), the curvature of the palate and the section of the infraorbital canal and the nasal cavities can be appreciated. Due to the selection of the threshold and the initial decision not to choose the turbinals, they cannot be analysed with these models, but differences are visible in the section of the infraorbital canal. This is flat in *B*. *fissidens*, subtriangular in *D*. *glyphodon* and *N*. *fodiens*, and subrounded in *B*. *brevicauda*, *C*. *russula* and *S*. *coronatus*

In the coronal view the curvature of the palate is more pronounced in the small shrews, *C*. *russula*, *S*. *coronatus* and also in *N*. *fodiens*, than in the others. However, in the sagittal section (Fig 8) *B*. *brevicauda* and *B*. *fissidens* also have a concave palate, even though *S*. *coronatus* shows the most marked concavity. *D*. *glyphodon* seems to be the straightest.

Both *B*. *fissidens* and *D*. *glyphodon* have mandibles that are curved in a labial direction, although this curvature in *B*. *fissidens* results in the top of the coronoid process being almost horizontal, whereas this is not observed in *D*. *glyphodon*. In the other species studied, the mandibles are straight; they are more open in *B*. *brevicauda* and *S*. *coronatus* than in *N*. *fodiens* or *C*. *russula*.

These models reveal that the occlusion is complete in *C*. *russula* but not in the soricines. In the soricines, there is a space between the antemolars when the molars are in occlusion.

### Example of an application: Measurements

Table 1 shows the results of the measurements taken in all the reconstructed “individuals”.

**Table 1:** Measurements.

	*Beremendia fissidens*	*Dolinasorex glyphodon*	*Blarina brevicauda*	*Neomys fodiens*	*Crocidura russula*	*Sorex coronatus*
RL	10.2	11.8	8.9	9.3	7.3	7.4
PF	1.4	1.5	1.4	1.4	0.6	0.9
UTL	10.9	12.4	9.7	9.4	8.1	7.6
UL	3.2	3.5	2.8	2.5	2.4	2.5
P4-M3	6.6	7.3	5.5	5.4	4.5	4.3
OW	3.9	4.3	4.8	3.2	3.5	2.3
IOW	5.4	5.7	5.5	4.1	3.8	3.6
AW	3.4	3.3	3.2	2.4	2.2	1.9
WM2	7.3	7.9	6.2	5.2	5.9	4.0
ZW	7.8	7.9	7.4	6.3	5.9	4.8
HM1	3.7	4.0	2.7	2.1	1.9	2.0
Hpost	5.6	4.7	4.1	3.3	3.2	3.1
HA2	2.8	2.8	2.2	1.8	1.6	1.4
CW	13.2	14.3	11.6	8.1	7.9	7.6
PGW	7.9	7.8	7.0	5.7	5.6	4.9
Hmand	6.8	7.1	5.8	4.8	4.6	4.1
αmand	45–120	60–100	70	35	45	65
PF/RL	0.14	0.13	**0.16**	0.15	0.08	0.12
UTL/RL	1.07	1.05	1.09	1.01	**1.11**	1.03
UL/RL	0.31	0.30	0.31	0.27	**0.33**	0.34
P4-M3/RL	**0.65**	0.62	0.62	0.58	0.62	0.58
OW/RL	0.38	0.37	**0.54**	0.34	0.48	0.31
IOW/RL	0.53	0.48	**0.62**	0.44	0.52	0.49
AW/RL	0.33	0.28	**0.36**	0.26	0.30	0.26
WM2/RL	0.71	0.67	0.70	0.56	**0.81**	0.54
ZW/RL	0.76	0.67	**0.83**	0.68	0.81	0.65
HM1/RL	**0.36**	0.34	0.30	0.23	0.26	0.27
Hpost/RL	**0.55**	0.40	0.46	0.35	0.44	0.42
HA2/RL	**0.27**	0.24	0.24	0.19	0.22	0.19
CW/RL	1.29	1.21	**1.30**	0.87	1.08	1.03
PGW/RL	0.77	0.66	**0.79**	0.61	0.77	0.66
Hmand/RL	**0.67**	0.60	0.65	0.52	0.63	0.55

Measurements of the reconstructions and measurements divided by the rostral length for comparison. Measurements are in mm except αmand, which is in degrees. The highest values for each parameter are in bold, and the lowest values are underlined.

In general, the largest measurements are from *D*. *glyphodon*, followed by *B*. *fissidens*. These are followed by *B*. *brevicauda* and *N*. *fodiens*, whose measurements are in some parameters larger in *B*. *brevicauda* and in others in *N*. *fodiens*. Finally, the smallest species are *C*. *russula* and *S*. *coronatus*, with *S*. *coronatus* generally having the smallest measurements.

These results are explained and discussed in the Discussion section.

## Discussion

### Reconstructions: Strengths and weaknesses

The models obtained are “constructed” with “pieces” from different individuals. This means that the resulting models are not the mean or a representative specimen of a species, and they should not be considered as such. They can be regarded as examples for studying general characteristics, comparing with other species, and providing an idea of the general shape.

There are other methods using quantitative techniques such as geometric morphometrics (see [55,56]). They are suitable alternatives to this way of reconstructing which can be better in many cases. However these techniques need good samples of reference [53].When 3D models and studies are scarce, manual restoration as presented here can be a semi-quantitative useful tool.

In fact, it is not the first time that *B*. *fissidens* has been reconstructed using fossils from different specimens. [31] performed 2D reconstructions using a fragmented skull and a mandible from different sites, one from Dmanisi (Georgia) and the other from Almenara-Casablanca (Spain). Although there may be geographical differences among the individuals of the same species from one locality to another, the general features were used to infer the general palaeobiology of the species.

In contrast, here all the fossils of each species are from the same site. In the case of *B*. *fissidens* all of them are from Sima del Elefante and from the same level (TE 9), whereas in the case of *D*. *glyphodon* all of them are from Gran Dolina and from two levels (TD5 and 6). This minimizes the geographical and temporal variation within the species. However, we have used fragments from four different individuals in each model, which results in a higher possibility that individuals with morphological differences may be mixed. We have tried to minimize this by using fragments with teeth with similar wear, which indicates a similar age [57], rescaling them to make them as comparable as possible, and we have assumed that there is no noticeable sexual dimorphism in soricines [58]. The scanning of concretions grants access to better-preserved material that in other cases would be impossible to access. In addition, the fact that the fragments used are digitalised adds reliability, since when the user is combining and transforming pieces, there is continuous reference to parts from other fossils. In the present case, for example, after combining and mirroring the upper teeth, scaling the model allows the occlusion with the lower teeth to be seen perfectly clearly. Conversely, having fragments of maxillae as a reference allows the aperture of the mandibles to be tested and adjusted. With teeth, by using transparencies we can adjust the position of each tooth in its corresponding alveolus. In addition, when mirroring one half of the maxilla, we have the benefit of a more poorly preserved, but extant other half, which serves as a reference for position. For these reasons it is easier to avoid human errors in reconstructions with 3D models than with drawings, even though the process is partially manual.

Apart from the accuracy associated with the provenience of the samples, reconstruction in 3D has many advantages. These include the ability to see characteristics in different views or to study parts or internal characters with only one model. To do this with 2D reconstructions it would be necessary to produce a new reconstruction for each view. In addition, with the same 3D model we can hide or show different parts of the model, such as the upper teeth, so at the click of a button we can see the arrangement of the teeth, the roots or whatever is required.

Further, 3D models also have all the advantages that apply to a normal model made from a single individual, enabling researchers to model chewing and other movements, to undertake engineering analysis and produce animations, under the assumption that the model is a reasonable approximation of a real individual. In S1 and S2 Movies two examples of animations produced with the reconstructions presented.

With the part of the skull reconstructed using parts from other species, such as *B*. *brevicauda* in the present case, the detailed data (e.g. measurements) need to be treated with caution, and we have thus decided to limit the conclusions we draw from our own reconstructions to general observations and relations with other anatomical parts. *B*. *brevicauda* is a species with characteristics similar to *B*. *fissidens* and *D*. *glyphodon* [32–35]. Although the extinct species are phylogenetically related [34], the relation with *Blarina* is not clear. [36] includes *Blarina* and *Beremendia* in the tribe Blarinini, but other authors, i.e. [59], argue only that they are part of the subfamily Soricinae However, although it is important to try to use species that are phylogenetically as close as possible, [37] demonstrated that the closest relation does not necessarily mean the best reference, that other physical characteristics have to be taken into account, and that in any case caution is due in basing assumptions on the parts reconstructed with these other species.

We have to note also that it is not indispensable that the surface of reference for the missing parts be an extant skull. The reference can be, for example, a predicted shape at the estimated fossil size made with geometric morphometrics taking into account the allometric variation [38–40].

Another, different possibility using the complete model is to make reconstructions from the living animal, as in [7] with a *Homo heidelbergensis* skull made from the deformation of a *Homo sapiens* skull. S2 Fig shows reconstructions of *B*. *fissidens* based upon different views of the model.

Bearing the above in mind, models can be used to make qualitative observations and study certain characteristics, as could be done with a single specimen. Especially, we can make more general observations if the models are scaled using mean values known for the species. Although they may contain features from different individuals, such models make it possible to take measurements and perform analyses that would otherwise be impossible.

### Specific study of the shrews modelled: Anatomy

#### Measurements

Taking RL as the reference to infer the size of the snout, the biggest snouts belong to *D*. *glyphodon*, followed by *B*. *fissidens*, as expected given that they are the “giant shrews”. *B*. *brevicauda* and *N*. *fodiens* have very similar RL, *N*. *fodiens* being slightly bigger, and the smallest are *C*. *russula* and *S*. *coronatus*. These data are in accordance with Hmand, which also indicates that the biggest snout belongs to *D*. *glyphodon*. According to these measurements, *N*. *fodiens* is smaller than *B*. *brevicauda*, which is consistent with their general size if inferred from weight [60]. RL is lower in *B*. *brevicauda* as a consequence of it being a pug-nosed shrew. It is important to bear in mind that the measurements give information only from the temporomandibular articulation to the proximal end, which corresponds to the incisors. The angle formed by the mandibles changes from the base to the top in *B*. *fissidens* and *D*. *glyphodon*. It is most heterogeneous and has the highest angle in *B*. *fissidens* and the lowest in *N*. *fodiens*.

As the species differ in size, the measurements have been divided by RL for comparison with one another, since this is the longest measurement of the bone. As the reference is to length, the proportions refer to the proportional width and height of the snout.

The main difference between *B*. *fissidens* and the other species is that the snout is more elevated than in the others, with HA2, HM1 and Hpost showing the highest values, giving a more vertical face to this species. These measurements are also variable, and there is a significant difference from the anterior to the posterior part, so the snout becomes very high in a short distance.

*B*. *brevicauda* stands out due to the relative width of the snout, all the relevant measurements being the relatively highest among the species studied (OW, IOW, AW, ZW, CW and PGW). CW and PGW, however, are very close to *Beremendia*, which is also very wide in the posterior part.

The main characteristic of *C*. *russula* is the high relative length of the dental series, which occurs because the unicuspids are not parallel to the rostral length and are bigger than in the other species. Another characteristic is that the snout is wider in the centre than in the posterior part.

*N*. *fodiens* is important because it has the lowest relative values for most of the parameters. It has the lowest values in the relative length of the tooth series (UL/RL, UTL/RL, P4-M3/RL), probably due to the similar shape of the tooth profile and the total length of the snout. At the same time, it is also the shrew with the narrowest and flattest snout and with the most parallel mandibles. In addition, it has the most regular nose, with smaller differences in height values.

Some of the values are similar to *S*. *coronatus*, which is also characterised by a narrow, but not such a flat snout.

*D*. *glyphodon* does not have extreme relative parameter values compared with the other species studied here. It is the biggest species, but the rest of its characteristics are intermediate, falling between *B*. *brevicauda* and *B*. *fissidens*.

#### Internal characteristics: Subnumerary teeth

One of the advantages of reconstructions based on microCT is that we can also use them to make observations of the interior of the model. In the specific case of the two studied soricids, we have identified for the first time that one of these species presents subnumerary teeth.

In the description of *D*. *glyphodon* in [32], it is said to have four upper unicuspids, but they are not present in the paratype, only the alveoli. Among the present *D*. *glyphodon* material, the A4 was not found either, so maxillae without this antemolar were selected. Although a rounded gap after A3 can be seen, in the microCT the alveolus was not found except on one side of one of the two skulls scanned. This indicates that the number of antemolars in *D*. *glyphodon* is variable.

[61] describe common anomalies related with teeth in shrews, and one of the most common (up to 5%) is subnumeracy of teeth, usually of the unicuspids. Although [62] do not encounter the anomaly in *Sorex araneus*, [63,64] say that in some individuals the last unicuspid is missing on one side or both. This phenomenon could be what is observed in *D*. *glyphodon*, since in one of the skulls there is only one A4. This indicates that the absence is not symmetrical, so this change does not seem to be the result of an adaptation to the environment or related to sexual dimorphism.

## Conclusions

The protocol described in the present paper have made it possible, for the first time, to reconstruct in 3D the anatomy of two small mammals that are systematically incomplete or fragmented in the fossil record. The 3D reconstruction of skulls belonging to extant and fossil shrews has enabled us to arrange the mandibles in their anatomical position, to orient them and facilitate comparisons among them, and to compare their internal structures. At present there is no other way of producing a complete reconstruction of fragmented small vertebrates with pieces from different individuals, except by means of 2D reconstructions using palaeoart. The advantages of our 3D models are firstly that with each step the insertion of each scaled element and the occlusion of teeth can be tested, and secondly that the final models allow researchers to do anything that is possible with a normal 3D model, such as rotating and measuring elements in any direction, seeing the interior without affecting the original pieces, and producing animations.

These reconstructions are also useful for activities of dissemination such as museum exhibits. These reconstructions allow a non-specialized audience to understand how extinct animals were.

The procedure in question combines the use of techniques such as the microCT scan, the 3D reconstruction of the pieces scanned, and the use of software developed for 3D design and printing, with the transformation and adaptation of fossil fragments from different individuals and a skull from another species, to create a model of the species studied. It involves the detailed use of software that is free and available on the internet and that allows the work to be done on a commercial computer, making this procedure accessible to a wide audience with different degrees of knowledge of 3D reconstructions.

## Supporting information

S1 Fig Effects of smoothing and reduction. Effects of smoothing and reduction in the shape and wireframe. Examples in a small object, the second upper antemolar of *Beremendia fissidens*, and in a large object, the mandible of *B*. *fissidens*
(TIF)

S2 Fig Reconstruction of the face of *Beremendia fissidens*. Reconstruction of the face of *Beremendia fissidens* using shots of the 3D reconstruction of the skull in different views. Artwork performed with water pencils.
(TIF)

S1 File Reconstruction of *Beremendia fissidens*. Final reconstruction of *Beremendia fissidens*. Reduced quality. Model units = mm.
(PDF)

S2 File Reconstruction of *Dolinasorex glyphodon*. Final reconstruction of *Dolinasorex glyphodon*. Reduced quality. Model units = mm.
(PDF)

S1 Movie Animation of the reconstruction of *Beremendia fissidens*. Animation in GIF format of the reconstruction of *B*. *fissidens* without the part inferred from *Blarina*.
(GIF)

S2 Movie Animation of the reconstruction of *Dolinasorex glyphodon*. Animation in GIF format of the reconstruction of *D*. *glyphodon* without the part inferred from *Blarina*.
(GIF)
