# Supplementary figures and images for: Protocol for the reconstruction of micromammals from fossils. Two case studies: The skulls of Beremendia fissidens and Dolinasorex glyphodon
Source: PLoS One. 2019 Mar 20;14(3):e0213174. doi: 10.1371/journal.pone.0213174 (PMC6426217; doi:10.1371/journal.pone.0213174)

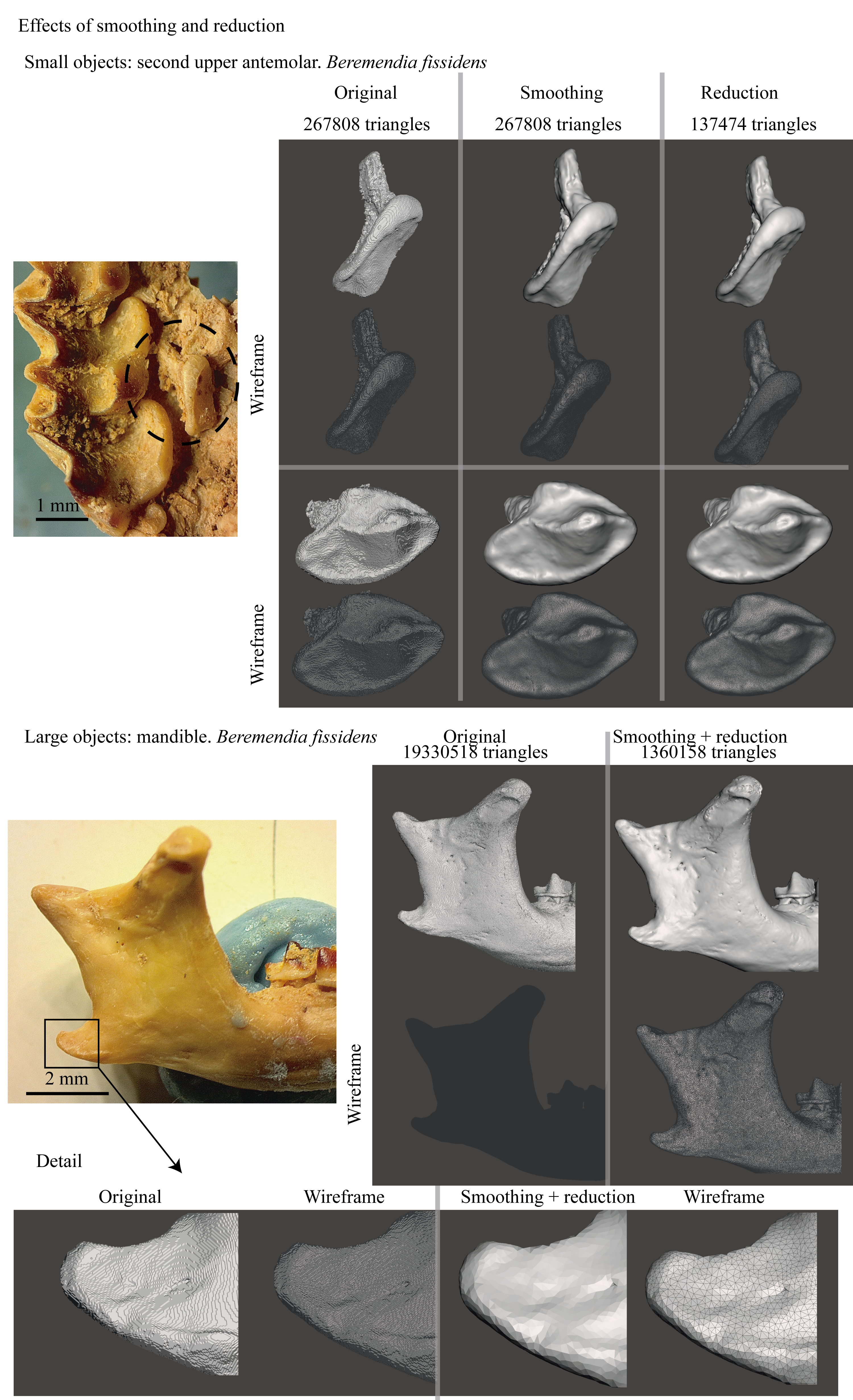

Supplement: S1 Fig — Effects of smoothing and reduction in the shape and wireframe. Examples in a small object, the second upper antemolar of Beremendia fissidens, and in a large object, the mandible of B. fissidens (TIF) [file pone.0213174.s001.tif]

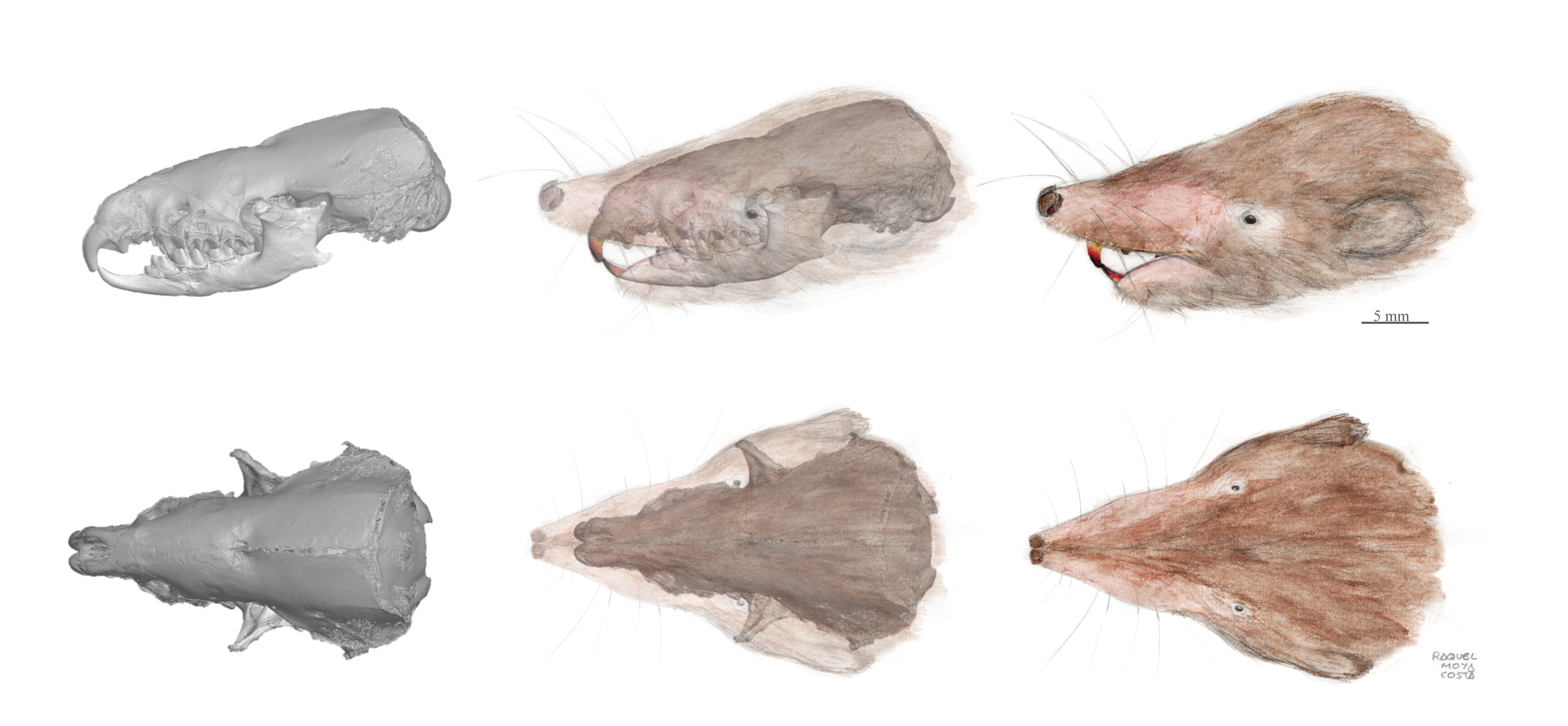

Supplement: S2 Fig — Reconstruction of the face of Beremendia fissidens using shots of the 3D reconstruction of the skull in different views. Artwork performed with water pencils. (TIF) [file pone.0213174.s002.tif]

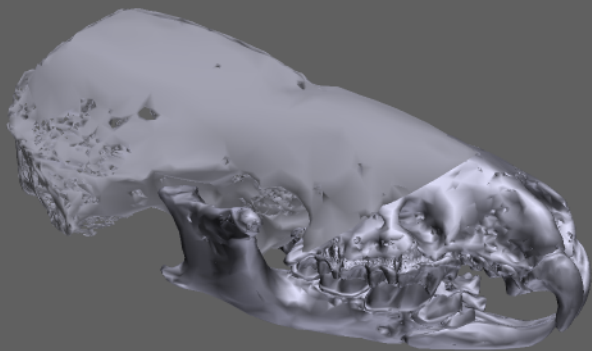

Supplement: S1 File — Final reconstruction of Beremendia fissidens. Reduced quality. Model units = mm. (PDF) [file pone.0213174.s003.pdf]

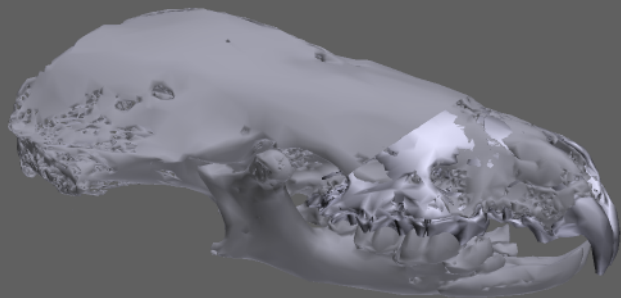

Supplement: S2 File — Final reconstruction of Dolinasorex glyphodon. Reduced quality. Model units = mm. (PDF) [file pone.0213174.s004.pdf]

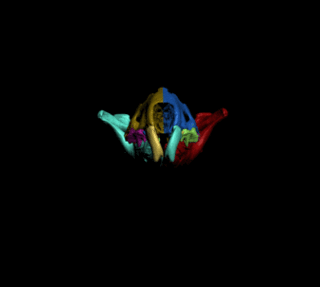

Supplement: S1 Movie — Animation in GIF format of the reconstruction of B. fissidens without the part inferred from Blarina. (GIF) [file pone.0213174.s005.gif]

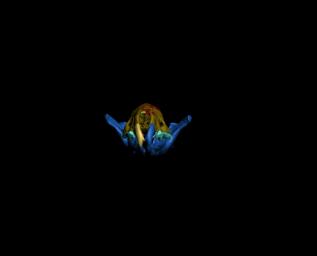

Supplement: S2 Movie — Animation in GIF format of the reconstruction of D. glyphodon without the part inferred from Blarina. (GIF) [file pone.0213174.s006.gif]
